# Evaluation of AT121 versus morphine on cortical neurons electrophysiology and dopamine concentrations in hippocampal cells

**DOI:** 10.1371/journal.pone.0347529

**Published:** 2026-04-20

**Authors:** Baraa E. Elawy, Chadi E. Soukkarieh, Abdul Q. Abbady, Shaza A. Allaham, Georges M. Deeb

**Affiliations:** 1 Department of Animal Biology, Faculty of Science, Damascus University, Damascus, Syrian Arab Republic; 2 Department of Molecular Biology and Biotechnology, Atomic Energy Commission of Syria (AECS), Damascus, Syrian Republic; 3 Department of Pharmacology, Faculty of Pharmacy, Damascus University, Damascus, Syrian Arab Republic; University of Nebraska Medical Center College of Medicine, UNITED STATES OF AMERICA

## Abstract

In order to achieve pain relief without associated tolerance and dependence risks of general opioids like morphine, researchers have designed AT121 as potent safe alternative. In this study, we evaluated the analgesic and neurochemistry effects of AT121, a bifunctional partial agonist at Mu and nociceptin/orphanin FQ peptide (NOP) receptors, compared to morphine in hippocampal neurons for the measurement of dopamine neurotransmitters concentration and action potential of cortical neurons isolated from newborn BALB/c mice. This helps us to predict and assess its success *in vivo* by detecting the effect of AT121 *in vitro*. This activates G0/Gi protein pathways while blocking the β-arrestin pathway, significantly delayed action potential generation, prolonged spike duration, and reduced amplitude, without altering firing thresholds or inducing tolerance over a two-hour window. In contrast, morphine has produced similar analgesic effects but with a higher risk of tolerance. Co-administration of AT121 and morphine improved these changes, whereas naloxone failed to reverse AT121’s effects, suggesting distinct receptor interactions. Dopamine quantification in hippocampal culture media revealed that morphine, alone or combined with AT121, markedly elevated extracellular dopamine, consistent with its reinforcing properties to morphine on analgesia. Notably, AT121 alone led to significantly lower dopamine levels compared to control, indicating a reduced risk of triggering reward-related pathways. Together, these findings highlight AT121 as a promising candidate for both acute and chronic pain management, and suggest its offering potent analgesia with a lower likelihood of tolerance and addiction following chronic opioid exposure.

## Introduction

Pain perception involves the transmission of nerve impulses. This process relies on sequential activation of voltage-gated sodium channels across the plasma membrane [1].

The therapeutic efficacy of opioids, in pain alleviation act by inducing hyperpolarization of neuronal cells via G-protein mediated pathway. This process involves the activation of Mu opioid receptors coupled with the G proteins and the activation of the β-arrestin pathway by G protein coupled receptor kinases (GRKs) phosphorylate. In addition, the cyclic adenosine monophosphate (cAMP) production is reduced by the G-proteins direct mediation. This cascade inhibits voltage-activated Ca2+ channels (VACCs), while G protein subunits, Gαi/o and beta/gamma β/γ, activate potassium channel [2–5]. Therefore, morphine’s analgesic effect stems from hyperpolarization via G protein-gated inwardly rectifying potassium (Kir) channel activation [3,6], which exhibits ion conduction during hyperpolarization [7]. Furthermore, the sodium channel modulates action potential characteristics in multipolar neurons within the nucleus accumbens (NAc) [8,9].

Opioid receptors are G protein-coupled receptors (GPCRs), comprise (Mu, Delta, and Kappa) and the naloxone-insensitive nociceptin/orphanin FQ (N/OFQ) receptor (NOP) [2]. Opioid is attributed to its ability to modulate nociceptive neurotransmission involve excitatory neurotransmitters within the synaptic cleft [10].

Opioid misuse, that of morphine, induces neuroadaptive changes coupled with dopamine release in reward and aversion circuits [11] including the cerebral cortex, hippocampus, ventral tegmental area (VTA), and NAc [12]. This effect can lead to a reduction of the GABA release, thereby disinhibits dopaminergic neurons and modulates inhibitory postsynaptic currents (IPSCs) [3].

Voltage-sensitive Nav1.6 sodium channel blockers exert a modulatory effect on neuronal activity by selectively suppressing glutamate secretion in hippocampal and influx GABA [8,13]. The hippocampus comprises (30%) excitatory pyramidal neurons, and (60%) granular cells involved in neurotransmitter release [1,14].

Chronic pain presents a significant global health challenge, affecting approximately 20% of the world’s population [15]. While mu-opioid receptor agonists remain the most potent analgesics available [16], the liabilities associated with their use, including withdrawal symptoms and the development of tolerance with chronic administration, exemplified by morphine [17], necessitate the development of safer alternatives. A promising candidate is AT121, a balanced dual agonist of both mu and (NOP) receptors [18]. This unique mechanism is hypothesized to attenuate pain signaling and delay tolerance development [10] without compromising digestive or respiratory function. Recent research, such as Ding et al. [11], has explored the effects of AT121 in comparison to morphine in preclinical models, including characterizing its functional activity at both mu and NOP receptors using Chinese hamster ovary cells.

Neuronal activity and response properties can be determined through electrical ion channels, by a voltage-sensing domain that responds to depolarization [19]. Therefore, the present study investigates the impact of AT121 on neuronal excitability and ionic currents in isolated cortical neurons from neonatal rats, utilizing whole-cell patch-clamp electrophysiology. Furthermore, we examine the effects of AT121 on dopamine in cultured hippocampal cells. This investigation aims to evaluate the potential of AT121 which generates effective analgesia by its efficiency on the hyperpolarization, while minimizing the released of dopamine, thereby reducing the risk of adverse effects commonly associated with traditional opioid analgesics. The comprehensive approach will provide valuable insights into the neurobiological mechanisms underlying AT121’s potential as a safer and more effective treatment for chronic pain.

## Methods

### Animal ethics and husbandry

This study, approved by the Animal Ethics Committee of the Damascus University (ID Number: Fos-200825–498), utilized three-month-old pregnant female BALB/C mice (n = 2, 35 g) sourced from the Molecular Biology and Biotechnology Department of Animals Housing Unit (AECS). Mice were maintained under controlled environmental conditions (28 ± 2°C, 40–60% humidity, 12-hour light/dark cycle) with ad libitum access to food and water. All procedures were conducted in strict accordance with the UK (Scientific Procedures) Act 1986 and European Union Directive 2010/63/EU guidelines for laboratory animal experimentation and accordance with ARRIVE guidelines [20].

### Cortical neurons and hippocampal cell culture preparation

Newborns’ BALB/C mouse (n = 15) heads were quickly and mercifully severed with a sharp scalpel. Then neurons from the cerebral cortices and hippocampal cells were isolated each aside from (1–3 hour) postnatal BALB/C mouse specimens in order to cultivate. Sterile microsurgical instruments (Bioair) were utilized to perform tissue dissections within a laminar flow hood (BIOAIR) under aseptic conditions. Following mechanical dissociation, enzymatic digestion with papain (Roche) was employed to process the tissues. The resulting cell suspension was then subjected to centrifugation through a bovine serum albumin (BSA) cushion using a refrigerated centrifuge (Sigma) to achieve purification. Subsequently, the cells were resuspended in Dulbecco’s modified Eagle’s medium (DMEM) with high glucose concentration (4.5 g/l) and L-Glutamine (Capricorn), supplemented with 10% fetal bovine serum (FBS, Sigma) and 1% penicillin/streptomycin (Cytogen, Germany) [14]. The cell cultures were then seeded into sterile 10 cm diameter culture dishes and maintained at 37°C in a humidified atmosphere of 5% CO2 (Biosan) for a period of four days prior to experimental procedures, including enzyme-linked immune sorbent assay (ELISA) analysis and electrophysiological recordings.

### Electrophysiological methods

Electrophysiological recordings were conducted on cortical neurons (n = 8 per group) isolated from neonatal rats (4–5 days post-culture) maintained in high-glucose DMEM. Borosilicate glass capillary electrodes (Harvard Apparatus LTD) with outer and inner diameters of 1.5 mm and 0.86 mm, respectively, were fabricated using a microelectrode puller (Narishige Group) and subsequently trimmed to a 1 μm diameter utilizing a Microforge MF-830 electrode trimmer equipped with a 525x magnification microscope (Narishige Group).

Cell-attached configurations were established on an INTRACEL vibration isolation table using a Nikon Eclipse Tί inverted research microscope. Seal resistances ranging from 3–8 GΩ were consistently achieved. The intracellular milieu was controlled using an electrode filling solution comprising (in mM): 105 K-gluconate, 30 KCl, 0.5 CaCl2, 2.4 MgCl2, 5 EGTA, 10 Hepes, and 5 Na2 ATP, 0.33 GTP-trisodium salt adjusted to an osmolality of 330 mOsm and a pH of 7.35 using ddH2O. Whole-cell patch-clamp access was facilitated by applying a brief negative pressure pulse to the recording pipette.

Following a 5-minute stabilization period after solution application, neuronal recordings were initiated and repeated after 2 hours. An access resistance of 60.7 MΩ was deemed acceptable for inclusion in the analysis. Whole-cell voltage-clamp and current-clamp recordings were performed at room temperature using an automated signal amplifier (Axon CNS Model Multiclamp 700B, Molecular Devices) held at a holding potential of −60 mV with a holding current of 7.4 pA. Stimulation was delivered at 2 kHz using an Axon CNS Digidata 1440A analog-to-digital converter. Resultant electrical impulses were recorded, and data were acquired using an Asus PC with Clampex 10.1 software (Molecular Devices).

Current-voltage relationships were assessed utilizing a 0.06 s voltage-clamp sweep protocol. To derive current density values, current amplitudes were normalized to cell capacitance (pF). The minimal direct current (DC) threshold required to elicit an action potential (AP) was determined through the analysis of a voltage derivative (dV/dT ≤ −10) using a specialized statistical software (Molecular Devices Clampfit 10.1). This software enabled signal analysis and saving, incorporating a low-pass Bessel filter (10 kHz) and a digitization frequency of 20 kHz.

To mitigate observational bias, the functional effects of the studied compounds were compared to an unbiased reference comparator, as outlined by a previously established method [2], allowing for the identification of similarities and differences in their effects [16].

### Drugs

The compounds investigated included AT121 (1 mg/ml, Cayman Chemical Company – Michigan,USA), a substance dissolved in DMSO (0.01% in sterile water), and Morphine Sulfate (10 mg/ml) (Darou PaKhsh – Tehran, Iran)., and acetylcholine (10 mg/ml, MARA Company – Bordeaux, France). The opioid receptor antagonist Naloxone HCl (0.4 mg/ml) (Taj Pharma – Fort Mumbai, India) was also employed.

The efficacy of the test substances on electrical measurements, specifically pain relief was assessed at a concentration of 10 µg/ml to compare effectiveness of the same concentration for all substances, according to our previous study. A dose-response curve with number of maximum activating concentrations, which was without toxic effect. Above that, morphine’s effect at this concentration did not show any effect on the cell viability at 72 h [17]. We add (10 µg/ml: AT121 equals to 21.6 nmol/ml) (10 µg/ml: morphine equals to 14.9 nmol/ml) (10 µg/ml: Naloxone HCl equals to 27.4 nmol/ml) (10 µg/ml: acetylcholine equals to 68.3 nmol/ml). This assessment relied on comparing the potential difference between the spike initiation point and the peak of its depolarization phase relative to the normal cellular state. Additionally, comparisons were made to a negative control consisting of acetylcholine (10 µg/ml). The activation threshold required for action potential generation, the time to first action potential, and the duration of total spike phases (from initiation to repolarization) were also quantified following compound application, both at 5 minutes and 2 hours post-application to evaluate the effects development of tolerance on potential [6].

### Dopamine quantification

Extracellular dopamine levels were determined in hippocampal cell cultures, hippocampus comprises (60%) granular cells involved in neurotransmitter release [1,14]. We chose using a commercially available sandwich enzyme-linked immune sorbent assay (ELISA) kit specific for rat dopamine (SUNLONG). Conditioned media, collected 30 minutes post-treatment with test compounds (10 µg/mL) or vehicle control from hippocampal cultures isolated from newborn mice, were analyzed. The assay principle involves the capture of dopamine by pre-coated antibodies, followed by detection with a horseradish peroxidase (HRP)-conjugated secondary antibody. Following incubation on a horizontal shaker (Heidolph), a chromogenic substrate (TMB) was added, and the resulting colorimetric signal, directly proportional to dopamine concentration, was quantified spectrophoto metrically at 450 nm using an ELISA plate reader (Thermoscientific). Dopamine concentrations were calculated (ng/mL) by interpolation from a standard curve generated using serial dilutions of dopamine provided in the kit, adhering to manufacturer’s instructions.

### Statistical analysis

Data analysis was performed using GraphPad Prism 5.0 software. Quantitative data are presented as mean ± standard deviation (SD), with ‘n’ representing the number of cells measured per subject. Statistical significance was assessed via ordinary One-way analysis of variance ANOVA followed by Tukey’s multiple pairwise comparisons or via Kruskal-Wallis test followed by Dunn’s multiple pairwise comparisons. Significance levels were defined as follows: *p < 0.05, **p < 0.01, ***p < 0.001, ****p < 0.0001.

The sample size was determined using GPower where the Power (1-β err prob), α error prob, actual power, and max. size is 0.90, 0.05, 0.91, and 56, respectively. Therefore, we have taken a total of cells (56): 7 plates and 8 cells which meets Cohen-ANOVA.

## Results

### Electrophysiological recordings

#### Investigating the impact of AT121 and morphine on action potential latency.

To investigate the effects of AT121 and morphine on neuronal electrical activity, we recorded action potentials in advanced neurons isolated from the cerebral cortex of new born BALB/c mice. In control cells, action potentials emerged with an average latency of 3.76 ± 0.33 ms. The addition of acetylcholine, a hyperpolarizing agent, resulted in a latency of 5.8 ± 0.24 ms, which was statistically significant compared to control neurons (P < 0.0001).

In contrast, Morphine (10 µg/ml) significantly delayed the onset of action potentials, with a latency of 6.7 ± 0.21 ms (P < 0.0001). Notably, AT121 (10 µg/ml) induced a delay in the onset of action potentials, with a latency of 7.59 ± 0.51 ms (P < 0.0001). When morphine and AT121 were co-administered, the latency of action potentials was prolonged to 8.9 ± 0.34 ms (P < 0.0001).

**Fig 1:**
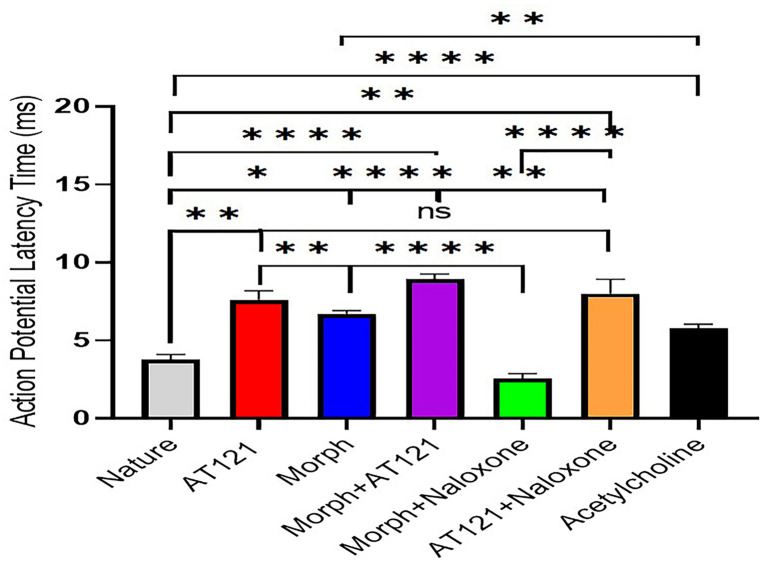
Effects of morphine, AT121, acetylcholine, and naloxone on action potential latency in newborn mouse cerebral cortex pyramidal cells. Substances were added at 10 µg/ml and latency measured after 5 minutes. Results show mean ± standard deviation (SD) from (n = 7 × 8 cells), with statistically significant differences indicated (*p < 0.05, **p < 0.01, ***p < 0.001, ****p < 0.0001).

To elucidate the underlying mechanisms, we examined the effect of naloxone (10 µg/ml) on morphine-induced changes in action potential latency. Naloxone completely blocked the effect of morphine, restoring the latency to near control values 2.45 ± 0.52 ms (P = 0.0001). Interestingly, naloxone enhanced the effect of AT121, resulting in an even greater delay in action potential onset, reaching 8.1 ± 0.89 ms (P < 0.0001), compared to control neurons (P = 0.6569) and neurons treated with AT121. These findings are illustrated in Fig 1 and S1 Table.

**Fig 2:**
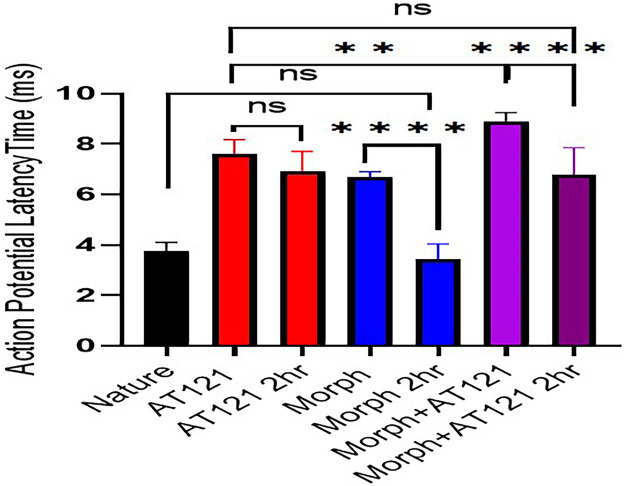
Effect of morphine and AT121 (10 µg/ml) on action potential latency in pyramidal cells from newborn mouse cerebral cortex. Data represent the mean ± standard deviation of (n = 7 × 8 cells), recorded 2 hours post-treatment. (*p < 0.05, **p < 0.01, ***p < 0.001, ****p < 0.0001).

Our results demonstrate that, following a two-hours incubation period, morphine’s influence on the delay in action potential onset time is abolished, with values returning to those observed in control cells 3.5 ± 0.86 ms (P = 0.9417). In contrast, AT121 maintains its effect on the delay in action potential onset time, exhibiting a significant delay 7.1 ± 0.82 ms (P = 0.3561) that is similar to the immediate effect seen upon addition. Notably, co-administration of AT121 and morphine guides to a remakable delay in action potential onset time 7.06 ± 1.05 ms (P < 0.0001) compared to 5 min after adding AT121 and morphine (matching the immediate post-addition values), (P = 0.9990), and solely 5 min after adding AT121, as illustrated in Fig 2 and S2 Table.

#### Assessing the combined impact of AT121 and morphine on neuronal spike duration.

**Fig 3:**
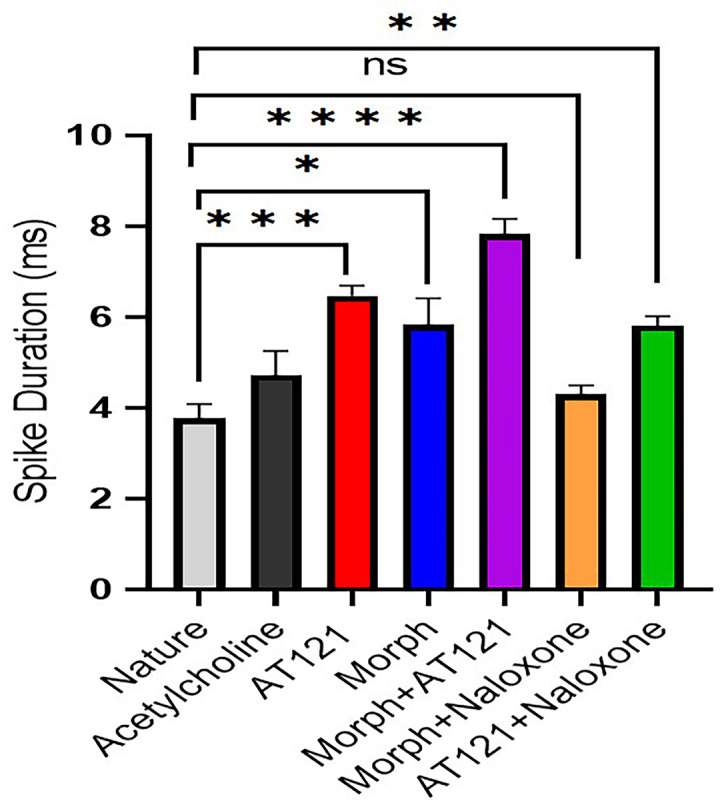
Modulation of action potential phases in newborn mice cerebral cortex pyramidal cells by morphine, AT121, acetylcholine, and naloxone. Mean total time ± SD is shown for each compound (10 µg/ml) added to the culture medium for 5 minutes. Statistical significance: (*p < 0.05, **p < 0.01, ***p < 0.001, ****p < 0.0001), (n = 7 × 8 cells).

We investigated the effects of AT121 and morphine on neurons cultures electrical activity isolated from newborn BALB/c mice. Specifically, we examined the time required to complete the stages of spike latency in neurons isolated from the cerebral cortex of these mice. In control neurons, the time required to complete the spike latency was 3.76 ± 0.31 ms. The addition of acetylcholine had a minimal and non-significant effect on latency time, increasing it to 4.72 ± 0.5 ms (P = 0.3638). In contrast, the application of AT121 at a concentration of 10 µg/ml significantly prolonged the latency time to 6.46 ± 0.2 ms (P < 0.0001). Morphine, at the same concentration, also increased the latency time, albeit to a lesser extent, to 5.82 ± 0.5 ms (P = 0.0025). The co-administration of both substances at the same concentration resulted in a latency time of 6.90 ± 0.34 ms (P < 0.0001). As shown in Fig 3 and S3 Table, the opioid receptor antagonist naloxone almost completely abolished the effect of morphine, reducing the latency time to 4.30 ± 0.1 ms (P > 0.9999). In contrast, naloxone did not reverse the effect of AT121, with the latency time remaining at 5.81 ± 0.2 ms (P = 0.0054).

**Fig 4:**
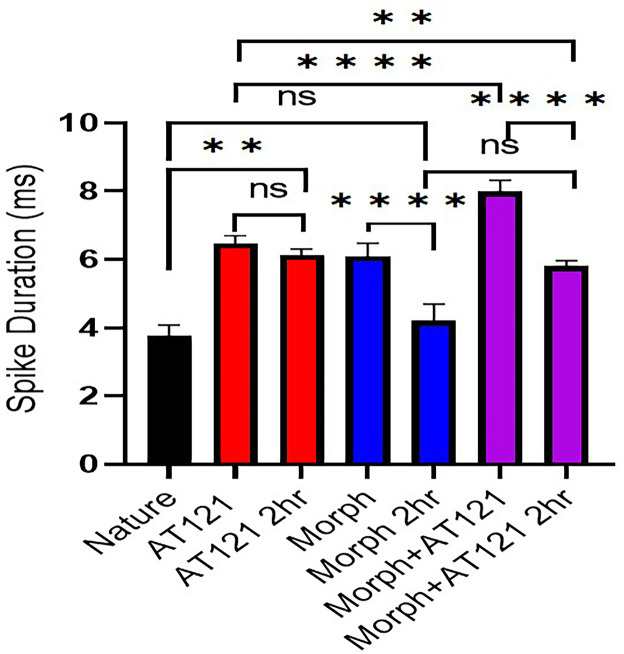
Morphine and AT121 effects on action potential duration in pyramidal cells. **2 hours after treatment.** mean ± SD total action potential time is shown for isolated from newborn mice cerebral cortex. (*p < 0.05, **p < 0.01, ***p < 0.001, ****p < 0.0001) (n = 7 × 8 cells).

The effects of morphine and AT121 on the latency phases of spike potentials in neurons cultures from newborn BALB/c mice were compared. As illustrated in Fig 4 and S4 Table, the latency period of spike potentials in response to morphine treatment returned to near-baseline levels 4.2 ± 0.4 ms (P = 0.1122) after a 2-hour incubation, similar to control cells. In contrast, AT121 maintained its significant effect on latency phases, with a mean duration of 6.1 ± 0.13 ms (P = 0.3721), even after 2 hours. Notably, a slight decrease in latency was observed in cells co-treated with morphine and AT121 compared to immediate post-addition values, with a mean duration of 5.8 ± 0.16 ms (P < 0.0001).

### Investigating the effects of AT121 and morphine on neuronal electrical activity and dopamine concentration

#### Determining the optimal stimulation threshold for neuronal response.

We examined the impact of adding AT121 and morphine on the threshold value of stimulation in neurons. Our results revealed that the excitatory threshold value in control neurons reached −67.62 ± 6.05 mV, following subtraction of the baseline value. Notably, the addition of acetylcholine, a hyperpolarizing substance, resulted in a significant decrease in the threshold value, reaching −137.44 ± 11.01 mV (P = 0.0008).

**Fig 5:**
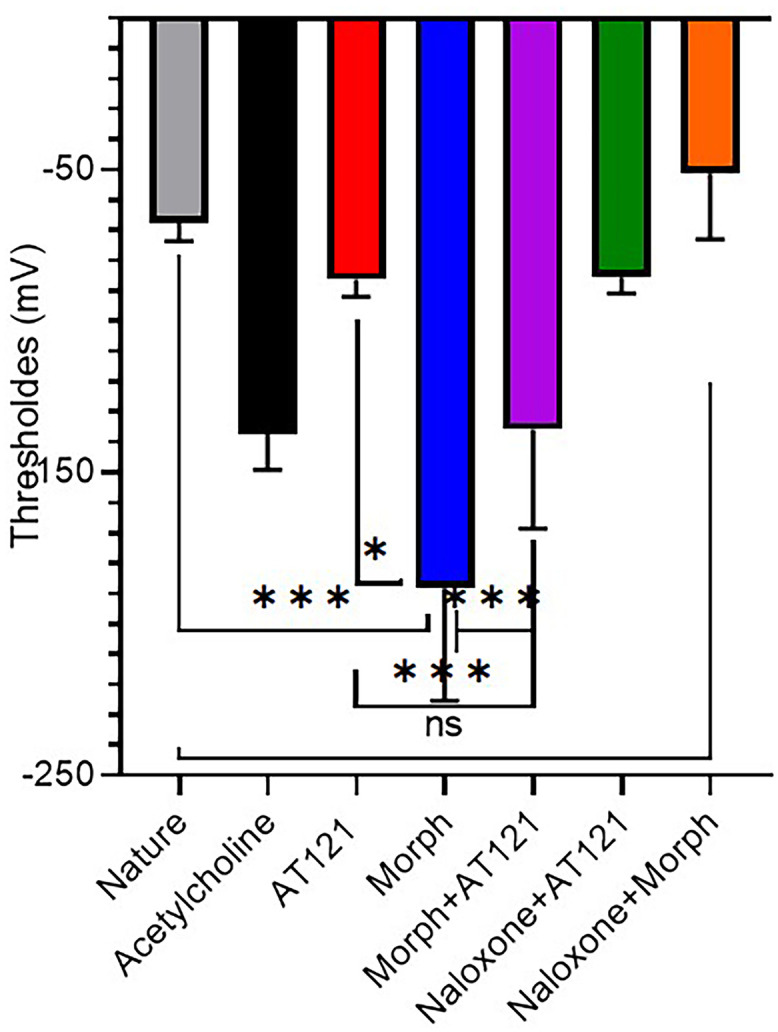
Effects of morphine, AT121, acetylcholine, and naloxone on stimulation threshold in pyramidal cells from newborn mice. The chart illustrates the mean ± standard deviation of the stimulation threshold in pyramidal cells isolated from the cerebral cortex of newborn mice, after 5-minutes of exposure to each of the specified compounds (10 μg/ml) in the culture medium (n = 7 × 8 cells) (*p < 0.05, **p < 0.01, ***p < 0.001, ****p < 0.0001).

Fig. 5 and S5 Table, illustrates the effects of morphine and AT121 on the threshold value. Morphine, at a concentration of 10 µg/ml, induced a profound decrease in the threshold value, reaching −188.16 ± 37.23 mV (P < 0.0001). In contrast, the addition of AT121 at a concentration of 10 µg/ml did not yield a statistically significant decrease in the threshold value, which remained at −86.41 ± 5.53 mV (P > 0.4374), similar to the control cells. The combined administration of morphine (10 µg/ml) and AT121 (10 µg/ml) resulted in a decrease in the stimulation threshold, although less pronounced than that observed with morphine alone, with a value of −135.86 ± 23.6 mV (P = 0.0044). The opioid receptor antagonist naloxone, at a concentration of 10 µg/ml, completely abolished the effect of morphine, returning the threshold value to −70.99 ± 24.11 mV (P > 0.9999). Furthermore, the co-administration of AT121 with naloxone led to a slight decrease in the threshold value, reaching −85.66 ± 5.37 mV (P = 0.7982), which approached the statistical differences observed between AT121 alone and control cells.

We compared the effects of AT121 and morphine on neuronal electrical activity. Specifically, we examined the threshold values after a 2-hour exposure to these substances.

**Fig 6:**
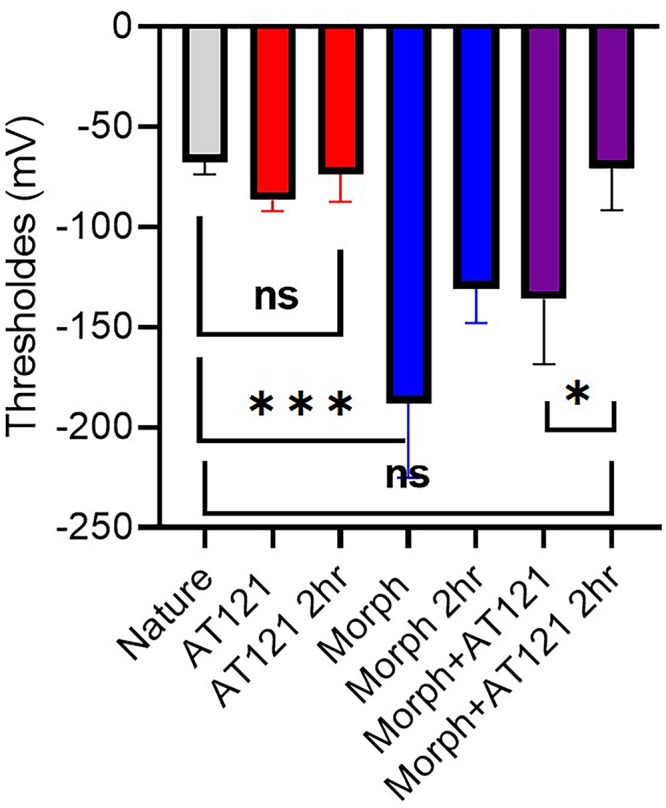
Impact of morphine and AT121 on pyramidal cell stimulation threshold. This figure illustrates the effect of morphine and AT121 (10 µg/ml) on pyramidal cells extracted from the cerebral cortex of newborn mice, 2 hours after addition (n = 7 × 8 cells). (*p < 0.05, **p < 0.01, ***p < 0.001, ****p < 0.0001).

As depicted in Fig 6 and S6 Table, our results show that morphine significantly increased the threshold value compared to its initial measurement, with a mean change of −137.02 ± 26.6 mV (P > 0.9999) compared to immediate morphine post-addition values, (P = 0.0023) compared to control neurons. In contrast, AT121 elicited a minimal increase in the threshold value, with a mean change of −73.5 ± 13.8 mV (P > 0.9999). Notably, the combined administration of morphine and AT121 resulted in a pronounced increase in the threshold value which is nearly equivalent to that observed in the control cells with a mean change of −70.4 ± 21 mV (P > 0.9999) compared to control neurons (P = 0.0401) that differentiate from the immediate Morphine and AT121 post-addition values. These findings provide valuable insights into the distinct effects of AT121 and morphine on neurons activity.

#### Study of the effect of adding AT121 and morphine on the amplitude of the action potential in neurons.

**Fig 7:**
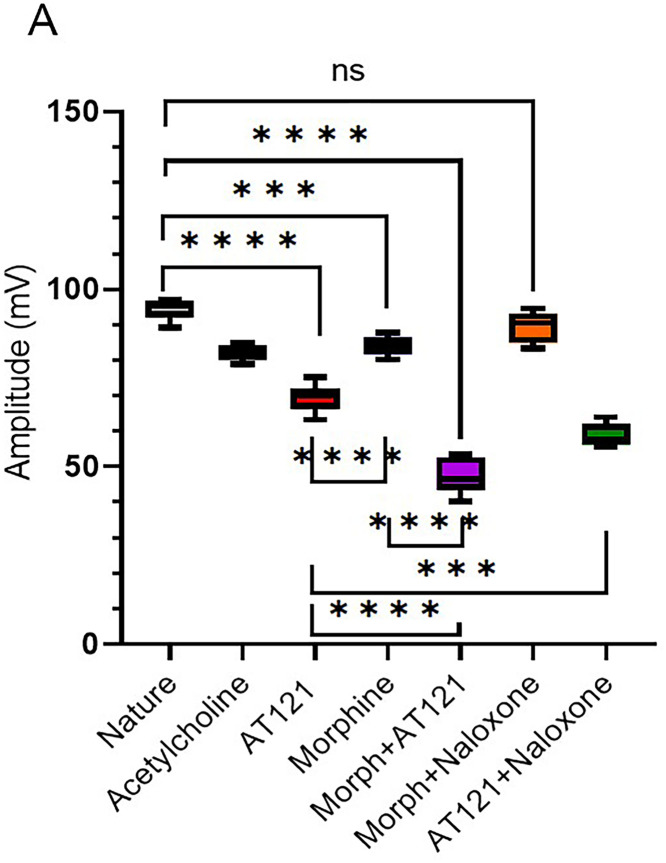
Modulation of action potential amplitude in pyramidal cells by morphine, AT121, acetylcholine, and naloxone. Mean ± SD amplitude is shown for 8 pyramidal cells isolated from newborn mouse cerebral cortex, after 5-minutes of exposure to each compound (10 μg/ml) in culture medium (n = 7 × 8 cells). (*p < 0.05, **p < 0.01, ***p < 0.001, ****p < 0.0001).

**Fig 8:**
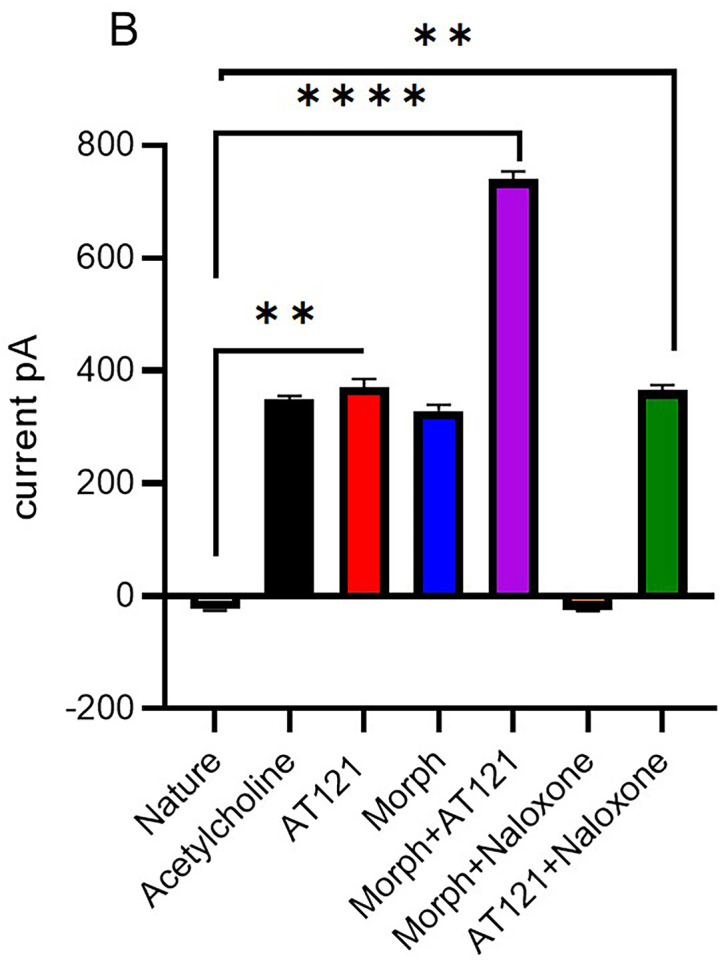
Modulation of cells membrane current in pyramidal cells by morphine, AT121, acetylcholine, and naloxone. Mean ± SD amplitude is shown for 8 pyramidal cells isolated from newborn mouse cerebral cortex, after 5-minutes of exposure to each compound (10 μg/ml) in culture medium (n = 7 × 8 cells). (*p < 0.05, **p < 0.01, ***p < 0.001, ****p < 0.0001).

**Fig 9:**
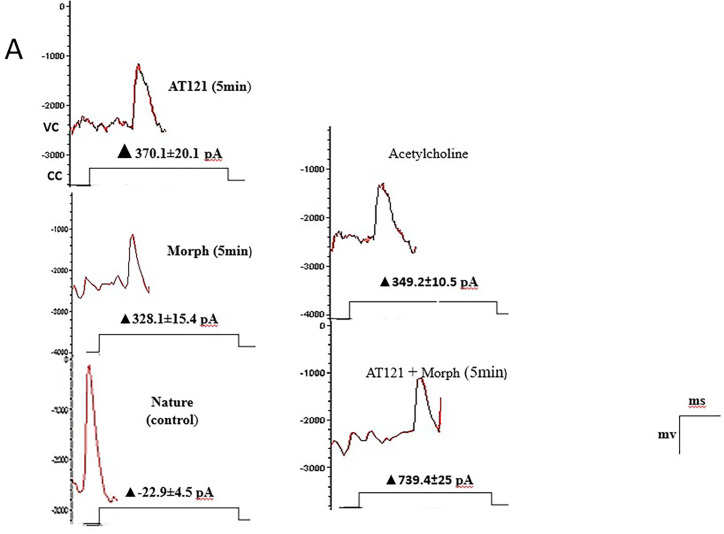
Modulation of action potential amplitude and membrane current in pyramidal cells from newborn mice cerebral cortex by morphine, AT121, and acetylcholine (10μg/ml). Measurements were taken 5 minutes after addition culture medium exposure (n = 7 × 8 cells). Signals were recorded in voltage-clamp mode at -60mV holding, with current measurements expressed in 0pA; While holding potential at 0mV in VC mode gives a + 10pA.

In the present study, we investigated the effects of AT121 and morphine on the amplitude of action potentials and cell membrane current in pyramidal neurons from newborn BALB/c mice cerebral primary cultures. Our results showed that electrical stimulation with a positive current led to depolarization of all stimulated cells, resulting in the generation of an action potential in the neurons 95.21 ± 3.46 mV removed from the cerebral cortex (control). The change in the current emitted from the cell membrane was recorded as ▲-22.9 ± 3.2 pA. To further elucidate the role of each substance on the electrical activity of the neurons, we evaluated the effect of AT121 and morphine on the amplitude of action potentials. The data illustrated in Fig 7, Fig 8 and Fig 9, and S7 Table and S8 Table demonstrate that the addition of acetylcholine at a concentration of 10µl/ml led to a slight decrease in the potential amplitude 82.11 ± 2.91 mV (P < 0.0001), accompanied by a change in the current value to ▲349.2 ± 5.10 pA.

Furthermore, we observed a more pronounced decrease in the potential amplitude when adding morphine at a concentration of 10µl/ml, resulting in an amplitude of 81.91 ± 2.68 mV (P = 0.0006) and a current change value of ▲328.1 ± 10.4 pA. Notably, the addition of AT121 at a concentration of 10µl/ml led to a more substantial decrease in the potential amplitude recording an amplitude of 67.36 ± 5.16 mV (P < 0.0001), accompanied by a change of ▲370.1 ± 12.1 pA. Moreover, we observed a greater decrease in the potential amplitude when adding morphine and AT121 together, as the spike amplitude was lower than its amplitude when adding morphine or AT121 alone, reaching a value of 47.08 ± 4.61 mV (P < 0.0001) compared to control neurons and a current change value of ▲739.4 ± 15 pA.

**Fig 10:**
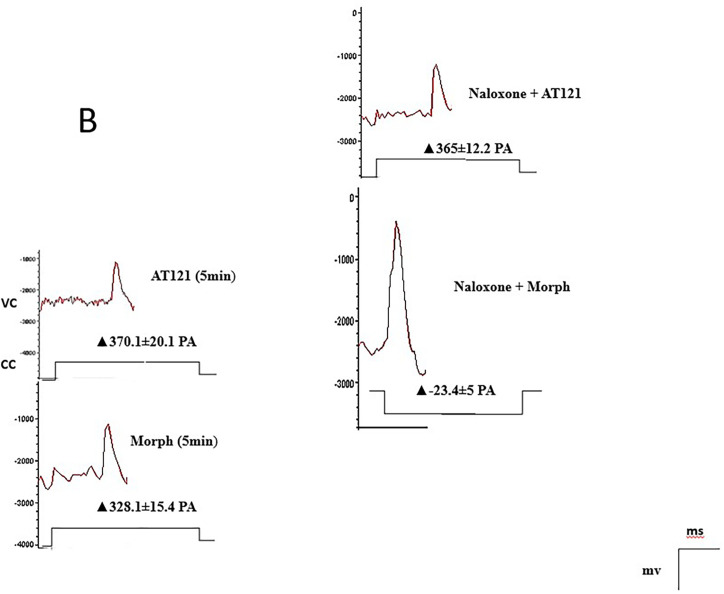
Modulation of action potential amplitude and membrane current in pyramidal cells from newborn mice cerebral cortex by morphine, AT121, and naloxone (10μg/ml). Measurements were taken 5 minutes after addition (n = 7 × 8 cells). Signals were recorded in voltage-clamp mode at -60mV holding, with current measurements expressed in 0pA; While holding potential at 0mV in VC mode gives a + 10pA.

Our study also investigated the effect of naloxone, an opioid antagonist, on the electrical activity of neurons. Notably, naloxone blocked the effect of morphine, as evident in Fig 7, Fig 8 and Fig 10. The potential amplitude recorded a value very close to the control cells 88.65 ± 4.37 mV (P > 0.2948), and the change in the current recorded a value close to its counterpart in the control cells ▲-23.4 ± 2.5 PA. In contrast, naloxone did not block the effect of AT121. A decrease in the value of the potential amplitude was observed, equivalent to 58.43 ± 3.31 mV (P < 0.0921) compared to the control, (P = 0.0003) immediate AT121 post-addition values, and a change in the current was very close to its change when adding AT121 alone ▲365.1 ± 7.62 PA.

Our findings provide valuable insights into the effects of AT121 and morphine on the electrical activity of neurons in the cerebral cortex cultures. The results obtained shed light on the potential therapeutic applications of these substances in the treatment at analgesia state.

**Fig 11:**
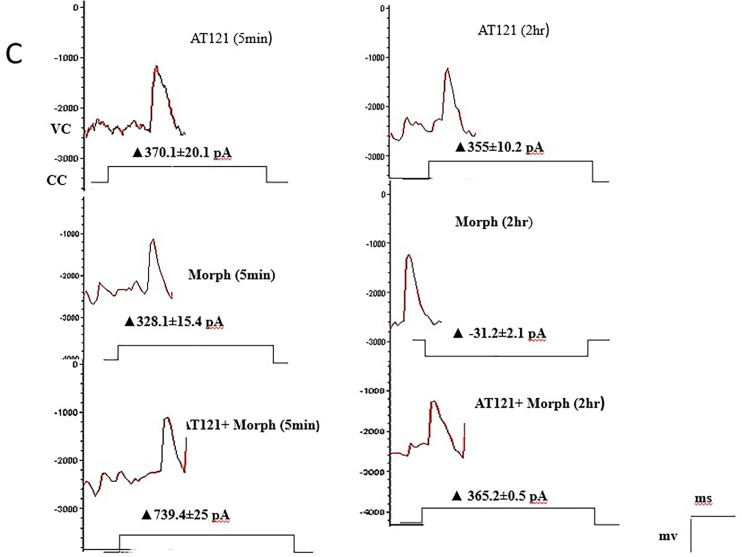
Modulation of action potential amplitude and membrane current in pyramidal cells from newborn mice cerebral cortex by morphine, AT121, and AT121 & morphine (10μg/ml). Measurements were taken 5 minutes after addition and 2 hours after culture medium exposure (n = 7 × 8 cells). Signals were recorded in voltage-clamp mode at -60mV holding, with current measurements expressed in 0pA; While holding potential at 0mV in VC mode gives a + 10pA.

**Fig 12:**
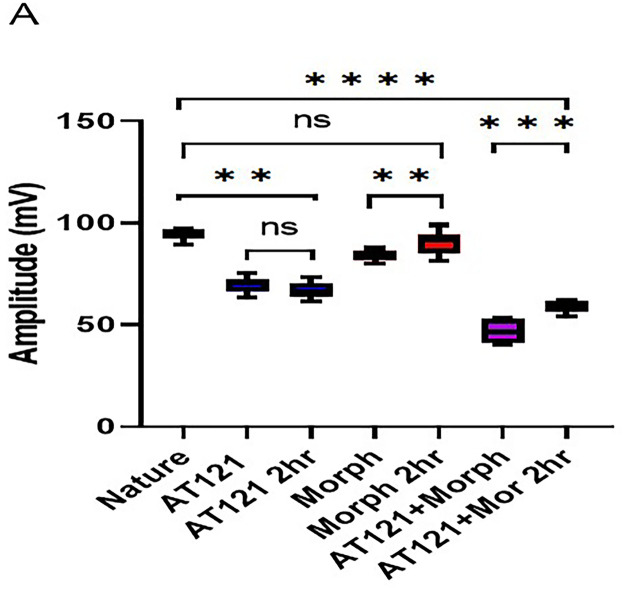
Morphine and AT121 modulation of action potential amplitude in pyramidal cell. Pyramidal cells extracted from newborn mice cerebral cortex. Examined after 2 hours of morphine and AT121 (10 µg/ml) exposure. (n = 7 × 8 cells). (*p < 0.05, **p < 0.01, ***p < 0.001, ****p < 0.0001).

**Fig 13:**
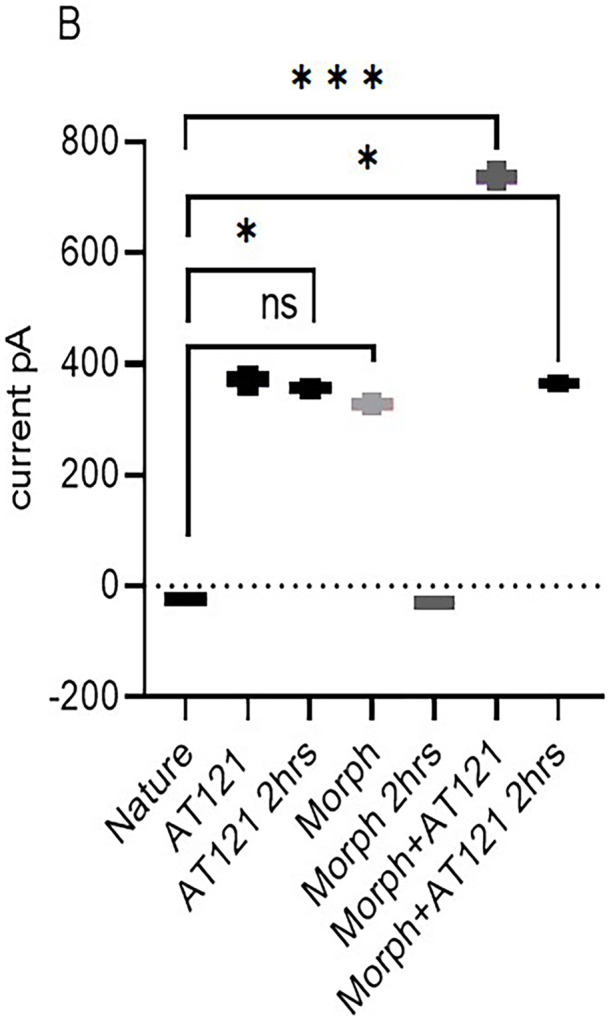
Morphine and AT121 modulation of cell membrane current in pyramidal cell. Pyramidal cells extracted from newborn mice cerebral cortex. Examined after 2 hours of morphine and AT121 (10 µg/ml) exposure. (n = 7 × 8 cells). (*p < 0.05, **p < 0.01, ***p < 0.001, ****p < 0.0001).

We investigated the effects of morphine and AT121 on neuronal electrical activity. Our results, as illustrated in Fig 11, Fig 12 and Fig 13, and S9 Table and S10 Table revealed distinct differences in the amplitude of action potentials and current changes in response to these two substances. Notably, the impact of morphine on action potential amplitude was short-lived, with the effect dissipating approximately two hours after administration. Specifically, the recorded amplitude was 90.04 ± 7.26 mV (P = 0.5416), accompanied by a decrease in the current emitted from the cell membrane, amounting to ▲-31.2 ± 2.1 pA. In contrast, AT121 sustained its amplitude-reducing effect, resulting in a depolarization value of 68.72 ± 4.3 mV (P = 0.0097) and a current change of ▲3555. ± 10.2 pA. Furthermore, when morphine and AT121 were co-administered, a differential response was observed. After a 2-hour incubation, a notable difference in potential and current change was recorded, akin to the effect of AT121 alone, with an amplitude of 58.75 ± 4.7 mV (P < 0.0001) compared to control neurons, (P = 0.0004) compared to immediate AT121 and Morphine post-addition values, and a current change of ▲365.2 ± 53. pA. These findings collectively suggest that AT121 exerts a more sustained influence on neuronal electrical activity compared to morphine 365.2 ± 5.3.

#### Investigating the effects of morphine and AT121 on dopamine levels.

**Fig 14:**
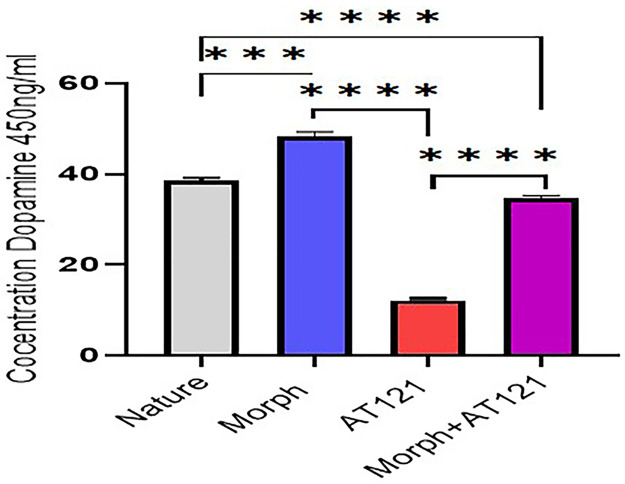
Effects of morphine and AT121 on dopamine concentration in hippocampal cell culture. Mean ± SD dopamine concentrations are illustrated after 30 minutes of treatment in culture dishes, measured using sandwich ELISA analysis at 450 nm. Statistical significance (*p < 0.05, **p < 0.01, ***p < 0.001, ****p < 0.0001) (Tukey’s multiple comparisons Test) (n = 4 × 3 culture dishes).

To elucidate the impact of morphine and AT121 on dopamine levels modulation, we quantified dopamine concentrations in the culture medium of hippocampal cells isolated from newborn BALB/c mice using sandwich ELISA analysis. Our results show that the dopamine concentration in the control group reached 38.753 ± 3.04 ng/ml (Fig 14, and Table 1 and S11 Table). Notably, a significant increase in dopamine concentration was observed 30 minutes after the addition of morphine at a concentration of 10 µg/ml, with a 124.87% elevation compared to the control group, reaching 48.391 ± 0.997 ng/ml (P < 0.0001). In contrast, the addition of AT121 at the same concentration (10 µg/ml) led to a substantial decline in dopamine concentration by 79.6% compared to the control group, resulting in a dopamine level of 12.38 ± 1.09 ng/ml (P < 0.0001). Furthermore, when both morphine and AT121 were added to the culture medium at the same concentration (10 µg/ml), we observed a decrease in dopamine concentration by 11.81%, reaching 34.56 ± 0.7 ng/ml (P = 0.0007) compared to the control group.

**Table 1:** Dopamine concentration in hippocampal cell culture medium: effect of morphine and AT121 (10 µg/mL) at 30 minutes.

Hippocampus	Morphine+AT121	AT121(10 µg/ml)	Morphine(10 µg/ml)	Nature(Control)
**Concentration ng/ml**	34.56 ± 0.7	12.38 ± 1.29	48.391 ± 0.997	38.753 ± 3.1
**Percentage %**	89.19%	31.94%	124.87%	100%

## Discussion

Our comparative evaluation of AT121 and morphine on neuronal electrical activity and dopamine modulation revealed distinct changes in electrical recordings. Notably, morphine, exposure of 5 minutes, exhibited a potent inhibitory effect on neuronal excitability, characterized by a reliable decrement in the amplitude of the action potential spike compared to control cells. This phenomenon can be attributed to morphine’s role in inhibiting sodium currents, as demonstrated by previous studies. Morphine has been shown to alter the steady-state voltage dependence of Na channels, rendering 50% of the channels inactive due to the hyperpolarization potential it induces [6,15]. Moreover, the addition of morphine leads to a reduction in stimulation threshold, resulting in an elevated rate of cell excitability [19], subsequent to the development of opiate-induced hyperalgesia (OIH) [15].

Post-administration of morphine after 2 hours on cellular activity was measured and observed characterized by the recovery of action potential lag time to normal levels and an increase in action potential amplitude upon stimulation, due to increased sodium influx and decreased potassium efflux. This is thought to be mediated by the reactivation of (cAMP) production, accompanied by an increase in the release of the GABA Transporter 1 (GAT-1) transmitter and in the presynaptic cleft and the subsequent release of GABA. The mechanisms underlying morphine tolerance may be linked to the phosphorylation of the carboxyl-terminal sites of (Mu-1B2, Mu-1C1) receptors. This phosphorylation event enhances the conversion of Gi/Go signals from the Mu receptor to Gs signals [2,21–24]. Furthermore, the prolongation of spike duration may be related to the depolarization potential induced by morphine through increased inhibition following transmission between the thalamic and dorsal striatal synapses [17].

Tolerance is often accompanied by oxidative stress in the hippocampus, neuroinflammation [25–27], also, levels of Mu receptor and mRNA of regulator of G protein signaling 17 (RGS17) in the prefrontal cortex increased [25,26], resulted from chromatin remodeling and enhanced transcription through histone lysine-tail acetylation, in genes within the VTA [6]. This is further complicated by an increase in the length, thickness and density of hippocampus microglia [15,27]. Given the critical role of the hippocampus, it was chosen for calibrating dopamine levels.

Overall, this diminished neuronal excitability in the presence of morphine is thought to be attributed to hyperpolarization [6], rendering the positive emitted membrane current. The majority of voltage-gated potassium channels (Kv) exhibit an open state when the membrane is depolarized [13]. Beta arrestin pathway is one of the intracellular cascades activated by opioid receptors activation, β-arrestin limits the effect of GABA interneurons [28], inhibits N-type voltage-gated calcium channels in VTA, which enables the release of dopamine in reward pathway, without effect on the nigrostriatal region [3,29]. In the context of our study, the increased dopamine observed in cultured hippocampus treated with morphine, compared to the control, corroborate our results. Additionally, the restoration of negative membrane current due to the reduced rate of potassium efflux from channels after 2 hours, are consistent with the development of tolerance.

In a notable contrast to morphine, our results demonstrate that AT121 exhibits a less pronounced depolarizing effect at an equivalent concentration. We propose that the enhanced analgesic efficacy of AT121 stems from its design as a partial agonist [11]. This co-activation of the dual receptors is hypothesized to augment the analgesic response via potentiation. Notably, data analysis reveals that AT121 induces more delay in action potential onset relative to morphine. we noticed a negligible decrease in the threshold value, suggesting that AT121’s action at NOP receptors involves blocking the β-arrestin pathway, thus reducing of tolerance [11,30–32]. This divergence in pathway selection may enable AT121 to maintain its analgesic potency while minimizing the negative impact [23]. Contrary to this, phosphorylation of the carboxyl-terminal sites of the Mu receptor has been shown to enhance analgesic activity without inducing tolerance, albeit without preventing the emergence of side effects [24]. When opioids are discontinued, a withdrawal syndrome often manifests, characterized by a plethora of neurological symptoms including nausea, anxiety, sweating, and cravings for the drug, ultimately leading to hypersensitivity to pain and loss of appetite [16]. Interestingly, research has demonstrated that deleting the β-arrestin gene can block its effectiveness, thereby enhancing the analgesic effect of morphine while weakening the induction of tolerance by disrupting the receptor’s sensitivity [33]. Furthermore, the β-arrestin pathway has been implicated in reducing the occurrence of respiratory and gastrointestinal side effects, as it is linked to cellular signals responsible for acute side effects [34].

Our study provides maintenance of the hyperpolarized effect was observed even after 2 hours of AT121 administration, with the electrical voltage required for depolarization and the delay in action potential onset remaining different from control. Moreover, the positive charge of the current emitted from the cell membrane persisted, suggesting the continued analgesic effect and potassium efflux from channels. The specific pathway engaged by this compound may modulate the threshold value and level, thereby delaying tolerance.

The activation of Go/Gi pathway by mu opioid receptors induces conformational changes in the receptor, thereby inhibiting cAMP formation. This pathway activates MAPK cascades [2], which modify opioid receptor trafficking (transcription, endocytosis etc), thus attenuate pain signals. Furthermore, the activation of NOP receptor modulates potassium channels, preventing the release of neurotransmitters including dopamine acetylcholine, serotonin, β-endorphin, norepinephrine, glutamate, and GABA neurotransmission [35]. Specifically, dopamine implicates in addiction and the reward pathway [32). The additional effect suggests AT121 has effect with tolerance is limited.

Exogenous administration of N/OFQ, an NOP agonist, has been shown to block dopamine in the limbic system [30,31]. So, AT121 does not stimulate dopamine release in the limbic system, avoids the tolerance, and (OIH), even upon chronic administration [11,13]. Our results, which demonstrated a decrease in dopamine concentration in neurons removed from the hippocampus, which is a part of limbic system composed of (60%) granular cells releasing dopamine, causing side effects such as tolerance and addition [14], support this notion. This is attributed to the fact that AT121 acts on Mu and NOP receptors [11].

The results might reveal a synergistic or additive efficacy effect when morphine and AT121 were co-administered to neurons at an equal concentration. Specifically, the latency of action potential onset was significantly prolonged. Furthermore, the amplitude of the action potential was reduced to a greater extent when both substances were combined. Notably, the electrical current generated during depolarization exhibited a greater positive charge when morphine and AT121 were co-administered, compared to the individual administration. These findings suggest that the co-administration of morphine and AT121 leads to an increased opening of potassium gates and restriction of sodium exit, resulting additional effect on the action potential. Consistently, the spike phase duration, was longer and stimulation threshold was higher when morphine and AT121 were co-administered, with the effect of AT121 appearing to raise the pain threshold despite the presence of the morphine effect However, this increase did not reach the same threshold value as observed with AT121 alone. The unique binding profile of AT121 is characterized by the formation of a hydrogen bond with Lys303 on the Mu receptor, unlike morphine and other addictive opioid, which interact with Tyr326, Asp147, and Tyr148. Additionally, Glu229, Lys303, and Trp318, previous studies have implicated these residues in addiction [11,33]. The molecular basis of this amplified effect can be attributed to the distinct binding sites of morphine and AT121 on the Mu receptor [29]. This difference in binding sites allows simultaneous modulation of the Mu receptor.

The examination of dopamine concentrations in hippocampal cell treated with both AT121 and morphine revealed similarity to those in control. This phenomenon can be attributed to the activation of the NOP receptor by AT121, which consequently blocks dopamine release [11]. In contrast, morphine leading to dopamine release [26]. The capacity of AT121 suggests its potential utility in treating addiction or achieving enhanced analgesic effects with reduced side effects.

Furthermore, AT121 demonstrates a superior efficacy compared to naloxone in managing addiction and mitigating morphine’s effects, primarily due to its ability to inhibit dopamine release. So, pharmacological channel blockade and the abolition of action potentials do not necessarily prevent the removal of inhibition of dopaminergic neurons in the VTA [3]. Instead, presynaptic VACCs and Ca2 + ingression play a crucial role in inhibiting presynaptic GABA release. The selective blockade inhibition of dopaminergic neurons, resulting in increased cumulative dopamine release. In contrast, naloxone administration is associated with systemic withdrawal symptoms due to the stimulation of norepinephrine release. While, opioids reduce adrenergic function by stimulating the Mu receptor in the locus coeruleus (LC) [28]. These findings collectively highlight the potential benefits of AT121 in mitigating morphine addiction and withdrawal, warranting further investigation into its therapeutic applications.

Adding naloxone blocks the morphine-induced effects. So, the action potential amplitude returned to values closely resembling those of control. Consistent with our results, naloxone significantly increased the excitatory threshold despite its co-administration with morphine, compared to the lower threshold when morphine was added alone. The emitted current exhibited an electrical charge very close to the emitted current in control cells, which may be attributed to its ability to block the effect on potassium gates [36]. In contrast, naloxone failed to completely block the effect of AT121, as evidenced by the delayed onset of action potentials, reduced spike amplitude, and increased positive charge of the membrane current compared to control cells and cells treated with both morphine and naloxone. This may be related to the fact that the analgesic effect of AT121 is mediated by its binding to both NOP and Mu receptors [11,18]. Recording potassium current stimulated by the induction of Mu by naloxone weakened the Kir channel current, consistent with its mechanism as a blocker [36]. This is consistent with the results of the emitted current value in our study.

Through binding dynamics AT121 appears to interact with the receptor via an alternative mechanism, as naloxone’s binding does not impede its activity. Morphine binds to the active site through three ligands, whereas AT121 binds to a single modified ligand on the side of the receptor. The agonist’s partial activity, which is balanced between the NOP and Mu receptors [18], may impose a single ligand on the Mu receptor, differing from the three ligands responsible for addictive given their shared effects. These findings confirm that naloxone cannot limit the inhibitory effect of AT121, which does not follow the traditional opioid pathway associated with addiction.

## Conclusion

In conclusion, The study’s findings on the electrical measurements of spike potentials and response times reinforce the potential of using AT121 for pain relief, circumventing the side effects associated with tolerance seen with opioids like morphine. Moreover, the reduction in dopamine levels in hippocampal cell cultures treated with AT121 indicates its potential utility in treating addition cases resulting from chronic exposure to Mu receptor agonists, which stimulate dopamine release through the reward pathway in the limbic system.

## Supporting information

S1 Table Effects of morphine, AT121, acetylcholine, and naloxone on action potential latency in neonate cerebral cortex pyramidal cells. Substances were added at 10 µg/ml and latency measured after 5 minutes.
(DOCX)

S2 Table Effect of morphine and AT121 (10 µg/ml) on action potential latency in pyramidal cells from neonate cerebral cortex, recorded 2 hours post-treatment. (DOCX)

S3 Table Modulation of action potential phases in newborn mice cerebral cortex pyramidal cells by morphine, AT121, acetylcholine, and naloxone (10 µg/ml) added to the culture medium for 5 minutes. (DOCX)

S4 Table Morphine and AT121 effects on action potential duration in pyramidal cells. 2 hours after treatment.
(DOCX)

S5 Table Effects of morphine, AT121, acetylcholine, and naloxone on stimulation threshold in pyramidal cells from newborn mice. The chart illustrates the mean ± standard deviation of the stimulation threshold in pyramidal cells.
(DOCX)

S6 Table Impact of morphine and AT121 on stimulation threshold on pyramidal cells extracted from the cerebral cortex of newborn mice, 2 hours after addition. (DOCX)

S7 Table Modulation of action potential amplitude in pyramidal cells by morphine, AT121, acetylcholine, and naloxone, after 5-minutes of exposure to each compound (10 μg/ml) in culture medium. (DOCX)

S8 Table Modulation of cell membrane current in pyramidal cells by morphine, AT121, acetylcholine, and naloxone., after 5-minutes of exposure to each compound (10 μg/ml) in culture medium. (DOCX)

S9 Table Morphine and AT121 modulation of action potential amplitude in pyramidal cell. Pyramidal cells extracted from newborn mice cerebral cortex Examined after 2 hours of morphine and AT121 (10 µg/ml) exposure.
(DOCX)

S10 Table Morphine and AT121 modulation of cell membrane current in Pyramidal cells extracted from newborn mice cerebral cortex. Examined after 2 hours of morphine and AT121 (10 µg/ml) exposure.
(DOCX)

S11 Table Effects of morphine and AT121 on dopamine concentration in hippocampal cells culture. (DOCX)
