# Supplementary material for: Evaluation of AT121 versus morphine on cortical neurons electrophysiology and dopamine concentrations in hippocampal cells
Source: PLoS One. 2026 Apr 20;21(4):e0347529. doi: 10.1371/journal.pone.0347529 (PMC13094985; doi:10.1371/journal.pone.0347529)
Supplement: S1 Table — Substances were added at 10 µg/ml and latency measured after 5 minutes. (DOCX) [file pone.0347529.s001.docx]

**Evaluation of AT121 Versus Morphine on Cortical Neurons Electrophysiology and Dopamine Concentrations in Hippocampal Cells.**

**Electrophysiological Recordings**

**1. Investigating the impact of AT121 and morphine on action potential latency**

|  | **Nature** | **Acetylcholine** | **AT121** | **Morph** | **Morph+AT121** | **Morph+Naloxone** | **AT121+Naloxone** |
| --- | --- | --- | --- | --- | --- | --- | --- |
| 1 | 3.3 | 5.3 | 7.5 | 6.7 | 9.3 | 2.5 | 9 |
| 2 | 4.1 | 6.1 | 8 | 6.8 | 8.6 | 3 | 7 |
| 3 | 3.9 | 5.8 | 6.7 | 6.9 | 9.2 | 2.5 | 8 |
| 4 | 3.7 | 5.7 | 6.9 | 7 | 8.7 | 2.7 | 8 |
| 5 | 4.2 | 5.9 | 8.4 | 6.5 | 8.7 | 2.3 | 9 |
| 6 | 3.4 | 6 | 7.5 | 6.4 | 9.4 | 3 | 7 |
| 7 | 3.5 | 5.9 | 7.7 | 6.5 | 8.5 | 2.2 | 7 |
| 8 | 4 | 5.5 | 8.1 | 6.8 | 8.9 | 2.1 | 9 |

Table S1: Effects of morphine, AT121, acetylcholine, and naloxone on action potential latency in **neonate** cerebral cortex pyramidal cells. Substances were added at 10µg/ml and latency measured after 5 minutes.
