# Supplementary material for: Evaluation of AT121 versus morphine on cortical neurons electrophysiology and dopamine concentrations in hippocampal cells
Source: PLoS One. 2026 Apr 20;21(4):e0347529. doi: 10.1371/journal.pone.0347529 (PMC13094985; doi:10.1371/journal.pone.0347529)
Supplement: S2 Table — (DOCX) [file pone.0347529.s002.docx]

**Evaluation of AT121 Versus Morphine on Cortical Neurons Electrophysiology and Dopamine Concentrations in Hippocampal Cells.**

**Electrophysiological Recordings**

**1. Investigating the impact of AT121 and morphine on action potential latency**

|  | **Nature** | **AT121** | **AT121 2hr** | **Morph** | **Morph 2hr** | **Morph+AT121** | **Morph+AT121 2hr** |
| --- | --- | --- | --- | --- | --- | --- | --- |
| 1 | 3.3 | 7.5 | 7.5 | 6.8 | 3.5 | 9.3 | 6.5 |
| 2 | 4.1 | 8 | 6 | 6.9 | 4 | 8.6 | 7.9 |
| 3 | 3.9 | 6.7 | 6.5 | 7 | 2.5 | 9.2 | 8.1 |
| 4 | 3.7 | 6.9 | 7.5 | 6.5 | 3.9 | 8.7 | 5.4 |
| 5 | 4.2 | 8.4 | 8 | 6.4 | 3.5 | 8.7 | 7.8 |
| 6 | 3.4 | 7.5 | 6 | 6.5 | 4.1 | 9.4 | 6.7 |
| 7 | 3.5 | 7.7 | 6.5 | 6.8 | 3.4 | 8.5 | 5.4 |
| 8 | 4 | 8.1 | 7.5 | 6.7 | 2.6 | 8.9 | 6.5 |

Table S2: Effect of morphine and AT121 (10µg/ml) on action potential latency in pyramidal cells from **neonate** cerebral cortex, recorded 2 hours post-treatment.
