# Supplementary material for: Evaluation of AT121 versus morphine on cortical neurons electrophysiology and dopamine concentrations in hippocampal cells
Source: PLoS One. 2026 Apr 20;21(4):e0347529. doi: 10.1371/journal.pone.0347529 (PMC13094985; doi:10.1371/journal.pone.0347529)
Supplement: S3 Table — (DOCX) [file pone.0347529.s003.docx]

**Evaluation of AT121 Versus Morphine on Cortical Neurons Electrophysiology and Dopamine Concentrations in Hippocampal Cells.**

**Electrophysiological Recordings**

**2. Assessing the combined impact of AT121 and morphine on neuronal spike duration**

|  | **Nature** | **Acetylcholine** | **AT121** | **Morph** | **Morph+AT121** | **Morph+Naloxone** | **AT121+Naloxone** |
| --- | --- | --- | --- | --- | --- | --- | --- |
| 1 | 4.2 | 4.9 | 6.1 | 6.5 | 7.4 | 4.2 | 6 |
| 2 | 3.8 | 5 | 6.3 | 5.7 | 8.2 | 4.2 | 5.9 |
| 3 | 4 | 4.5 | 6.8 | 5.304001 | 7.5 | 4.4 | 6.1 |
| 4 | 3.7 | 5 | 6.5 | 5.2 | 7.8 | 4 | 5.8 |
| 5 | 3.2 | 3.5 | 6.4 | 5.8 | 7.9 | 4.2 | 5.5 |
| 6 | 3.5 | 5 | 6.6 | 5.232 | 7.6 | 4.6 | 5.9 |
| 7 | 3.7 | 5 | 6.7 | 6.16 | 8.4 | 4.5 | 5.6 |
| 8 | 4 | 4.9 | 6.3 | 6.71 | 7.8 | 4.3 | 5.7 |

Table S3: Modulation of action potential phases in newborn mice cerebral cortex pyramidal cells by morphine, AT121, acetylcholine, and naloxone (10µg/ml) added to the culture medium for 5 minutes.
