# Supplementary material for: Evaluation of AT121 versus morphine on cortical neurons electrophysiology and dopamine concentrations in hippocampal cells
Source: PLoS One. 2026 Apr 20;21(4):e0347529. doi: 10.1371/journal.pone.0347529 (PMC13094985; doi:10.1371/journal.pone.0347529)
Supplement: S5 Table — The chart illustrates the mean ± standard deviation of the stimulation threshold in pyramidal cells. (DOCX) [file pone.0347529.s005.docx]

**Evaluation of AT121 Versus Morphine on Cortical Neurons Electrophysiology and Dopamine Concentrations in Hippocampal Cells.**

**Electrophysiological Recordings**

**Investigating the effects of AT121 and morphine on neuronal electrical activity and dopamine concentration**

**3. Determining the optimal stimulation threshold for neuronal response**

|  | **Nature** | **Acetylcholine** | **AT121** | **Morph** | **Morph+AT121** | **Naloxone+AT121** | **Naloxone+Morph** |
| --- | --- | --- | --- | --- | --- | --- | --- |
| 1 | -59.3499 | -162.18 | -74.8399 | -202.78 | -141.44 | -85.45 | -52.6299 |
| 2 | -69.1099 | -138 | -89.7 | -144.49 | -114.58 | -83.92 | -15.29 |
| 3 | -66.3599 | -129.44 | -86.49 | -118.76 | -95.97 | -89.14 | -70.3399 |
| 4 | -76.74 | -140.05 | -85.65 | -217.42 | -188.13 | -96.45 | -52.63 |
| 5 | -72.47 | -132.38 | -93.92 | -218.34 | -144.18 | -82.64 | -39.91 |
| 6 | -59.3499 | -140.76 | -89.65 | -212.53 | -175.62 | -86.41 | -84.37 |
| 7 | -66.94 | -134.35 | -84.8399 | -184.84 | -112.75 | -78.92 | -60.95 |
| 8 | -70.64 | -122.38 | -86.23 | -206.43 | -114.28 | -82.41 | -35.29 |

Table S5: Effects of morphine, AT121, acetylcholine, and naloxone on stimulation threshold in pyramidal cells from newborn mice. The chart illustrates the mean ± standard deviation of the stimulation threshold in pyramidal cells.
