# Supplementary material for: Evaluation of AT121 versus morphine on cortical neurons electrophysiology and dopamine concentrations in hippocampal cells
Source: PLoS One. 2026 Apr 20;21(4):e0347529. doi: 10.1371/journal.pone.0347529 (PMC13094985; doi:10.1371/journal.pone.0347529)
Supplement: S6 Table — (DOCX) [file pone.0347529.s006.docx]

**Evaluation of AT121 Versus Morphine on Cortical Neurons Electrophysiology and Dopamine Concentrations in Hippocampal Cells.**

**Electrophysiological Recordings**

**Investigating the effects of AT121 and morphine on neuronal electrical activity and dopamine concentration**

**3. Determining the optimal stimulation threshold for neuronal response**

|  | **Nature** | **AT121** | **AT121 2hr** | **Morph** | **Morph 2hr** | **Morph+AT121** | **Morph+AT121 2hr** |
| --- | --- | --- | --- | --- | --- | --- | --- |
| 1 | -59.3499 | -74.8399 | -94.8399 | -202.78 | -118.34 | -141.44 | -62.91 |
| 2 | -69.1099 | -89.7 | -59.7 | -144.49 | -117.023 | -114.58 | -72.37 |
| 3 | -66.3599 | -86.49 | -68.71 | -118.76 | -139.09 | -95.97 | -23.85 |
| 4 | -76.74 | -85.65 | -59.65 | -217.02 | -138.032 | -188.13 | -82.91 |
| 5 | -72.47 | -93.92 | -67.49 | -218.34 | -155.38 | -144.18 | -72.37 |
| 6 | -59.3499 | -89.65 | -72.7801 | -212.03 | -102.78 | -175.62 | -73.85 |
| 7 | -66.94 | -84.8399 | -94.49 | -184.84 | -144.49 | -112.75 | -94.49 |
| 8 | -70.64 | -86.23 | -70.96 | -206.23 | -130.96 | -114.28 | -80.96 |

Table S6: Impact of morphine and AT121 on stimulation threshold on pyramidal cells extracted from the cerebral cortex of newborn mice, 2 hours after addition.
