# Supplementary material for: Evaluation of AT121 versus morphine on cortical neurons electrophysiology and dopamine concentrations in hippocampal cells
Source: PLoS One. 2026 Apr 20;21(4):e0347529. doi: 10.1371/journal.pone.0347529 (PMC13094985; doi:10.1371/journal.pone.0347529)
Supplement: S7 Table — (DOCX) [file pone.0347529.s007.docx]

**Evaluation of AT121 Versus Morphine on Cortical Neurons Electrophysiology and Dopamine Concentrations in Hippocampal Cells.**

**Electrophysiological Recordings**

**Study of the effect of adding AT121 and morphine on the amplitude of the action potential in neurons**

| **Naloxone+Morph** | **Naloxone+AT121** | **Morph+AT121** | **Morph** | **AT121** | **Acetylcholine** | **Nature** | **Amplitude**  **(A)** |
| --- | --- | --- | --- | --- | --- | --- | --- |
| 94.621 | 61.4136 | 53.5801 | 86.6602 | 71.11 | 82.5319 | 97.12 | 1 |
| 89.78 | 56.2899 | 52.4899 | 80.23 | 67.3267 | 84.95 | 99.83 | 2 |
| 91.21 | 55.6 | 40.2899 | 82.1455 | 75.1987 | 83.83 | 92.954 | 3 |
| 83.28 | 64.1 | 44.58 | 85.1987 | 69.58 | 80.48 | 89.64 | 4 |
| 92.78 | 57.119 | 45.58 | 84.1455 | 70.2899 | 78.95 | 95.8 | 5 |
| 85.28 | 58.1 | 47.3267 | 80.4899 | 63.3267 | 81.97 | 95.65 | 6 |
| 82.31 | 53.12 | 48.61 | 76.15 | 61.72 | 84.21 | 98.5 | 7 |
| 90.01 | 61.01 | 44.22 | 80.4 | 60.4 | 79.98 | 92.2 | 8 |

Table S7: Modulation of action potential amplitude in pyramidal cells by morphine, AT121, acetylcholine, and naloxone, after 5-minutes of exposure to each compound (10 μg/ml) in culture medium.
