# Supplementary material for: Evaluation of AT121 versus morphine on cortical neurons electrophysiology and dopamine concentrations in hippocampal cells
Source: PLoS One. 2026 Apr 20;21(4):e0347529. doi: 10.1371/journal.pone.0347529 (PMC13094985; doi:10.1371/journal.pone.0347529)
Supplement: S8 Table — (DOCX) [file pone.0347529.s008.docx]

**Evaluation of AT121 Versus Morphine on Cortical Neurons Electrophysiology and Dopamine Concentrations in Hippocampal Cells.**

**Electrophysiological Recordings**

**Study of the effect of adding AT121 and morphine on the amplitude of the action potential in neurons**

| **Naloxone+Morph** | **Naloxone+AT121** | **Morph+AT121** | **Morph** | **AT121** | **Acetylcholine** | **Nature** | **Current**  **(B)** |
| --- | --- | --- | --- | --- | --- | --- | --- |
| -23.7 | 365.1 | 739.4 | 328.1 | 370.1 | 349.2 | -22.3 | 1 |
| -26.5 | 355.6 | 758.2 | 338 | 390 | 351 | -24.1 | 2 |
| -20.6 | 364.2 | 725.4 | 340 | 350.01 | 345.5 | -25.7 | 3 |
| -21.9 | 377.01 | 744.7 | 317.5 | 359.9 | 345 | -26.1 | 4 |
| -22.6 | 372 | 726.2 | 314 | 385 | 340 | -20.9 | 5 |
| -26.4 | 369.1 | 733.16 | 333.2 | 369 | 355 | -18.5 | 6 |
| -19.9 | 363.2 | 732.12 | 333.5 | 365.2 | 359.1 | -19.6 | 7 |
| -25.6 | 354.8 | 756.1 | 321.05 | 372.01 | 349.5 | -26.6 | 8 |

Table S8: Modulation of cell membrane current in pyramidal cells by morphine, AT121, acetylcholine, and naloxone., after 5-minutes of exposure to each compound (10 μg/ml) in culture medium.
