# Supplementary material for: Evaluation of AT121 versus morphine on cortical neurons electrophysiology and dopamine concentrations in hippocampal cells
Source: PLoS One. 2026 Apr 20;21(4):e0347529. doi: 10.1371/journal.pone.0347529 (PMC13094985; doi:10.1371/journal.pone.0347529)
Supplement: S9 Table — Pyramidal cells extracted from newborn mice cerebral cortex Examined after 2 hours of morphine and AT121 (10 µg/ml) exposure. (DOCX) [file pone.0347529.s009.docx]

**Evaluation of AT121 Versus Morphine on Cortical Neurons Electrophysiology and Dopamine Concentrations in Hippocampal Cells.**

**Electrophysiological Recordings**

**Study of the effect of adding AT121 and morphine on the amplitude of the action potential in neurons**

| **Morph+AT121 2hr** | **Morph+AT121** | **Morph 2hr** | **Morph** | **AT121 2hr** | **AT121** | **Nature** | **Amplitude**  **(A)** |
| --- | --- | --- | --- | --- | --- | --- | --- |
| 61.3301 | 53.5801 | 96.12 | 86.6602 | 62.2899 | 71.11 | 97.12 | 1 |
| 59.64 | 52.4899 | 98.9 | 80.23 | 73.5801 | 67.3267 | 99.83 | 2 |
| 54.01 | 40.2899 | 96.99 | 82.1455 | 69.58 | 75.1987 | 92.954 | 3 |
| 62.01 | 44.58 | 89.64 | 85.1987 | 68.2899 | 69.58 | 89.64 | 4 |
| 59.7 | 45.58 | 81.53 | 84.1455 | 66.01 | 70.2899 | 95.8 | 5 |
| 56.2 | 47.3267 | 86.431 | 80.4899 | 64.58 | 63.3267 | 95.65 | 6 |
| 51.8 | 48.61 | 79.2 | 76.15 | 72.5 | 61.72 | 98.5 | 7 |
| 65.1 | 44.22 | 91.5 | 80.4 | 73.01 | 60.4 | 92.2 | 8 |

Table S9: Morphine and AT121 modulation of action potential amplitude in pyramidal cell. Pyramidal cells extracted from newborn mice cerebral cortex Examined after 2 hours of morphine and AT121 (10 µg/ml) exposure.
