# Supplementary material for: Evaluation of AT121 versus morphine on cortical neurons electrophysiology and dopamine concentrations in hippocampal cells
Source: PLoS One. 2026 Apr 20;21(4):e0347529. doi: 10.1371/journal.pone.0347529 (PMC13094985; doi:10.1371/journal.pone.0347529)
Supplement: S10 Table — Examined after 2 hours of morphine and AT121 (10 µg/ml) exposure. (DOCX) [file pone.0347529.s010.docx]

**Evaluation of AT121 Versus Morphine on Cortical Neurons Electrophysiology and Dopamine Concentrations in Hippocampal Cells.**

**Electrophysiological Recordings**

**Study of the effect of adding AT121 and morphine on the amplitude of the action potential in neurons**

| **Morph+AT121 2hr** | **Morph+AT121** | **Morph 2hr** | **Morph** | **AT121 2hr** | **AT121** | **Nature** | **Current**  **(B)** |
| --- | --- | --- | --- | --- | --- | --- | --- |
| 365.5 | 739.4 | -31.1 | 328.1 | 356.1 | 370.1 | -22.3 | 1 |
| 364.2 | 758.2 | -33.6 | 338 | 363.1 | 390 | -24.1 | 2 |
| 355.9 | 725.4 | -29.2 | 340 | 342 | 350.01 | -25.7 | 3 |
| 372.5 | 744.7 | -31.8 | 317.5 | 344.9 | 359.9 | -26.1 | 4 |
| 370.1 | 726.2 | -28.4 | 314 | 359.5 | 385 | -20.9 | 5 |
| 362.3 | 733.16 | -33.2 | 333.2 | 367.55 | 369 | -18.5 | 6 |
| 369.2 | 732.12 | -33.6 | 333.5 | 346.1 | 365.2 | -19.6 | 7 |
| 362.1 | 756.1 | -29.4 | 321.05 | 365.5 | 372.01 | -26.6 | 8 |

Table S10: Morphine and AT121 modulation of cell membrane current in Pyramidal cells extracted from newborn mice cerebral cortex .Examined after 2 hours of morphine and AT121 (10 µg/ml) exposure.
