# Supplementary material for: Evaluation of AT121 versus morphine on cortical neurons electrophysiology and dopamine concentrations in hippocampal cells
Source: PLoS One. 2026 Apr 20;21(4):e0347529. doi: 10.1371/journal.pone.0347529 (PMC13094985; doi:10.1371/journal.pone.0347529)
Supplement: S11 Table — (DOCX) [file pone.0347529.s011.docx]

**Evaluation of AT121 Versus Morphine on Cortical Neurons Electrophysiology and Dopamine Concentrations in Hippocampal Cells.**

**4. Investigating the effects of morphine and AT121 on dopamine levels**

| **Dopamine Concentration** | **Nature** | **AT121** | **Morphine** | **Morphine+AT121** |
| --- | --- | --- | --- | --- |
| 1 | 35.549 | 12.386 | 49.394 | 35.3 |
| 2 | 38.959 | 11.092 | 48.38 | 34.5 |
| 3 | 41.751 | 13.675 | 47.4 | 33.9 |

Table S11: Effects of morphine and AT121 on dopamine concentration in hippocampal cells culture.
